# Comparative Influence of Certain Localities on Health, Particularly in the Production of Consumption

**Published:** 1880-10

**Authors:** H. P. Gatchell

**Affiliations:** Atlanta, Ga.


### COMPARATIVE INFLUENCE OF CERTAIN LOCALITIES ON HEALTH, PARTICULARLY IN THE PRODUCTION OF CONSUMPTION.

By H. P. GATCHELL, M.D., Atlanta, Ga.

While studying the vital statistics of the census of 1850, which gives the mortality of counties as well as of States, I was impressed with the evidence of the salubrity of Northeast Georgia. This impression was confirmed by subsequent investigation, and when my own failing health compelled me to seek a more congenial climate, I sought Northeast Georgia.

As the railroad has become a necessity of modern civilization, I shall include in this essay only those counties that are cut by the Atlanta and Charlotte Air-Line Railroad, with the single exception of Rabun county (soon to

*Diseases of the bladder and urethra in women, page 195.

be penetrated by a branch road,) which joins the others on the north. For convenience of reference, I shall designate the region thus defined, as the Northeast Plateau.

Rabun county lies on the southeast slope, and at the base of the Blue Ridge, which towers along its northern border, as does the less lofty Tallulah Range along its southern. The general altitude of Rabun, exclusive of mountain ranges, is over two thousand feet.

The other counties, Habersham, Hall, Gwinnett, DeKalb and Fulton, are on the Chattahoochee Ridge. This ridge, which is traversed by the Air-Line Road, has a general altitude of twelve or fourteen hundred feet, rising to eighteen hundred in Habersham, back to Toccoa, and to eleven hundred at Atlanta, in Fulton county.

In Habersham is a beautiful cascade, Toccoa Falls, where a sheet of water descends unbroken from a rampart of rock one hundred and ninety feet in height; and in Rabun are the wild cataracts and tremendous cliffs of Tallulah, in the midst of a noble amphitheatre, surrounded by magnificent mountain scenery.

Rabun is a very mountainous county; Habersham is considerably so, but much less than Rabun. The other counties are hilly and not mountainous.

According to the Smithsonian chart, the isothermal lines of this part of Georgia are parallel to the Chattahoochee Ridge, which has a general northeast and southwest direction, the summer mean being about 76°, and that of winter about 44° Fah. Gainesville fifty-three, and Toccoa ninety-three miles northeast of Atlanta, have even a warmer summer than has the latter place. Mount Airy, eighty miles northeast, and with an altitude five hundred feet greater, reported for the summer of 1879, and for the entire year, the same temprature as that of Atlanta; though ordinarily both summer and winter at Mount Airy are a little cooler than at Atlanta.

It will assist the reader's apprehension of the climate to consider that the isolation of 76° extends as far north as Washington City in the east, and nearly to the northern boundary of Kansas in the west, while that of 44° is found in northwest Texas; though on the Atlantic coast it reaches Albemarle Sound in North Carolina.

The temprature of Rabun county is about 70° in summer, and 40° in winter. The former occurs in New York in the east, and in Minnesota in the west; the latter in Alabama and Mississippi, as well as in the highlands of Georgia.

It is proper to remind the reader that the temperature of Rabun was not taken by a representative of the Signal Service, and that the thermometers of this service are placed at an altitude where they indicate a temperature several degrees lower in summer and higher in winter, than do instruments nearer the earth's surface.

The climate of the Northeast Plateau, like that of the whole country east of the Sierra Nevada, is subject to extremes in temperature. But for a given mean these extremes are much less in the South than in the North.

Thus, Washington, with a summer of 76° has a winter of 36° and Manhattan, Kansas, with the same summer mean, has a winter of 29°.

Albany, New York, with a summer of 70° has a winter of 29°, and Fort Snelling, Minnesota, with a summer a little warmer has a winter of 16°.

Santa Fe, New Mexico, with a summer of 70°, has a winter of 30°, while Denver and Colorado Springs, with a warmer summer than Santa Fe, have a colder winter; though, in consequence of the exceeding dryness of the air in the Rocky Mountain region, heat and cold are felt much less than in more humid sections.

Places, also, along the Allegany Mountains in a higher latitude than Rabun, have a considerable less equable temperature than has the mountain region of Northeast Georgia. Lewisburg, Virginia, near the White Sulphur Springs, at about the same altitude as Rabun county, has a summer of 72°, and a winter of 33°

But it is in examining single extremes of temperature that the greater equableness of Southern stations is more distinctly seen. For while at Southern the thermometer seldom rises as high in summer as in Northern stations, it does not fall nearly so low in winter. And the further north, at least this side of the Arctic Circle, the greater is the range of the thermometer between the two extremes of temperature.

The extremes of temperature thus far noted, are for Atlanta 90° and 3°, and for Rabun 95° and 6°, and though the line of observation is not long, there have occurred during it, a period of extraordinary heat and one of extraordinary cold; so that, though equal extremes may occur within a year, it is equally probable that they may not occur again for several years.

Within the period of these observations a depression of temperature occured so severe that vegetation suffered, as it had not before on the Plateau for more than forty years.

OFFICE

In explanation of the greater depression at Atlanta than in Rabnn county, I would add that the observations of Atlanta extend over a longer period than in Rabun, those for the latter including only two years. But one of those two years was distinguished by an unusual degree of cold, during which the thermometer indicated 7° at Atlanta and 6° in Rabun. Nor is it singular that the mercury should fall scarcely lower in Rabun than in Fulton county, the depression of temperature at the higher often being less than at the lower level. And this is because of the tendency, in case of a sudden depression of temperature, of the warm air to rise as it is displaced by the cold air from above.

To that tendency are due those thermal belts which occur on mountain sides, in which the latest spring frost is ten or twelve days earlier, and the earliest autumn frost ten or twelve days later than at the general level of the country, the contrast being still more marked between mountain sides, or hill sides, and valleys.

These thermal belts, often escaping entirely the influence of frosts and suffering only in case of a freeze, afford desirable areas for fruit growers. They are found everywhere among the Alleghanies, but especially on the southern slope of the Blue Ridge. The observations in Rabun county were made in Rabun Gap, within high and steep mountain walls, where both the extreme heat and extreme cold are likely to be greater than in the open country.

Let no one be surprised at a reduction of temperature in Atlanta to 3°. Forty-five years ago it fell to 2° below zero at Augusta, Georgia, and to 6° above at Fort Moultrie, in Charleston, South Carolina, harbor. And it has been noted at 11° at Fort Barrancas, near Pensacola, and at Fort King, still further south in Florida.

Comparing Washington City and St. Louis, places having the same summer mean as that of Atlanta, we find at Washington a maximum of 103° above and a minimum of 14° below zero, and at St. Louis a maximum of 108° above and of 25° below zero. But it should also be borne in mind that the observations at these two places are for long periods.

Comparing Albany, New York, and Brookside, Iowa, each having the mean summer of Rabun, with the latter, we find for the former a maximum of 97° above and a minimum of 23° below zero, and for the latter a maximum of 105° above, and of 35° below.

The same characteristic, that of great extremes of temperature, marks the Rocky Mountain region, the Plains and the country bordering on the Plains It is because of their nearness to the Plains that such extremes appear in Minnesota, Iowa and the States just west of the Mississippi and Missouri Rivers. From British America into Texas, also into New Mexico, Arizona and Southeast California, the extremes are everywhere great and sudden for the latitude. Reduction of temperature from the British line into Texas of 30° or 40° in a few hours is not uncommon.

In respect to extremes of temperature, this region resembles the North, with greater and more sudden vicissitudes than those of the North this side of the Mississippi.

*Table of Mean and Extreme Temperature.*

| | Sum'er. | Winter. | Maxim'm. | Minim'm, |
|---|---|---|---|---|
| Atlanta, Ga | 76 | 44 | 90 | 3 |
| Washington, D. C | 76 | 36 | 103 | —14 |
| St, Louis, Mo | 76 | 33 | 108 | —25 |
| Ft. Leavenworth. Kansas | 75 | 29 | 105 | —30 |
| Rabun County, Ga | 70 | 40 | 95 | 3 |
| Albany, N. Y | 70 | 26 | 97 | —23 |
| Brookside, Iowa | 70 | 18 | 105 | —35 |
| Ft. Snelling, Minn | 71 | 16 | 100 | —37 |

*Mean and Extreme Temperature in Interior of Texas.*

| | Sum'er. | Winter. | Maxim'm. | Minim'm. |
|---|---|---|---|---|
| Ft. Davis | 76 | 46 | 107 | —15 |
| Ft. Richardson | 82 | 47 | 109 | —10 |
| Ft. Gramam | 82 | 49 | 112 | 5 |
| Ft. Mason | 82 | 51 | 114 | 11 |
| Austin | 82 | 51 | 107 | 6 |
| San Antonio | 84 | 53 | 108 | 14 |

The extremes are not so great on the coast of Texas; but there the increased humidity nearly or quite compensates, rendering the same degree of heat more relaxing and of cold more chilling.

For the sake of emphasizing the greater extremes of Texas, I give two points, both interior, and in the same latitude, one in Texas and one in Georgia:

| | Summer. | Winter. | Maxim'm. | Minim'm. |
|---|---|---|---|---|
| Ft. Richardson, Texas | 82 | 46 | 107 | .10 |
| Augusta Arsenal, Ga | 80 | 48 | 103 | 2 |

The observation for Augusta Arsenal are for a long period, for Ft. Richardson for a very short one. With a long period at Ft. Richardson, the contrast would, no doubt, be still greater. In the table containing Northern and Southern, Eastern and Western points, the comparison would be more just if the periods did not differ. But no length of period could affect the general conclusions.

To bring this out more distinctly, I will add two other points, Ft. Simpson, latitude 62°, British America, and Ft. Gibson, latitude 35°, Indian Territory, near the Arkrnsas line.

| | Summer. | Winter. | Maxim'm. | Minim'm. |
|---|---|---|---|---|
| Ft. Simpson | 59 | 11 | 104 | .54 |
| Ft. Gibson | 79 | 40 | 116 | .20 |

With this illustration of the extremes of temperatures at the North, and near the plains, I conclude the subject of temperature

*Humidity.*—Humidity tends to aggravate the effects of both heat and cold, with the former to produce inflammatory and febrile affections, and with the latter to cause colds and other pulmonary diseases, and, among them, consumption.

In supervising the census of North Georgia, I have had ample confirmation of an observation, which I long since made, of the increased ratio of mortality in narrow valleys. Such valleys, especially in mountainous countries, are hotter by day and colder by night than more open areas. The nights, too, are characterized by dense fogs, which contribute to render the cold night air more chilling. What with the confined air and the heat by day, and the chilly dampness of night, the mortality from both fevers and consumption is increased.

On the other hand, excessive dryness is not without its disadvantages. The glare of the almost perpetual sunshine, in rainless or nearly rainless regions, causes irritation of the nervous system, resulting in paralysis or insanity, as is notably the case in California.

There is a tendency, also, as I have frequently noticed, in bright sunshine to cause, with sensitive persons, irritation of the liver, often attended by nausea and vomiting of bile And many persons incur a headache from exposure to the sun.

So far as I can judge, a mean humidity of about 60° (100° being the point of saturation), other conditions being equal, is that which is most favorable to the healthy actions of all the organs and systems of the body.

This is the mean for Atlanta, which, for all the places this side of the almost rainless plains, reported by the Signal Service last year, was the lowest except at one other point. That grain and tobacco can be stored here with an exemption from mold, that obtains in no other Southern city, has long been known.

To the invalid it is pleasant to be able, on most evenings, to sit out of doors without danger of his clothes being saturated with dew. It is seldom, except when rain is gathering, that enough dew lies on the grass, before late bedtime, to wet the hand that is passed along it.

In presenting these points of superiority, I do not wish to be understood as attempting to describe a perfect climate. Such does not exist outside of some tropical Highlands.

We have some unpleasantly hot summer, and some unpleasantly cold winter days. We have, too, some protracted winter rains. But these are soon absorbed by the porous soil, leaving, in most cases, the ground fit to walk on within a short time after the rain ceases.

Violent storms are rare at any season of the year, much more so than I have been accustomed to witness in the Northwest.

On the whole, the climate is eminently desirable. It is comparatively free from extreme heat or extreme cold. It has a predominance of clear, bright skies, with an inspiring dryness and purity of atmosphere.

Major DeForest, favorably known as soldier and author, was military commandant of a district in upper South Carolina after the war. From an article which he wrote for *Harper's Monthly*, I quote language which is still more applicable to the Northeast Plateau :

"In this same land, numberless water privileges send their ungathered riches to the sea, and the earth is crowded with underground places of mineral wealth. The climate, too, is unrivaled. The summer heat in Greenville was rarely too great for walking, its highest point being usually 84 ; while the winter brought at the worst two or three falls of snow, which melted in two or three days. Neither in Europe nor [anywhere] along the shores of the Mediterranean have I found a temperature which, during the year round, was so agreeable and healthful. You can see what it is in the remarkable stature of the men, and in the height, fullness of form and beauty of the women. My impression is, from Maryland down to the north of Georgia is a paradise for the growth of the human plant. If bodily comfort and intellectual pleasures existed there, I should advise all New England to emigrate to it."

I shall not attempt in this popular sketch to use the terms climate and climatic, in relation to health, with scientific precision. I shall include under these terms, indirect as well as direct influences, those that act by modifying habits of life, or by contributing to the development of noxious vegetable or animal forms, as well as those that affect directly the human system. In fine, I

shall include all meteorological influences direct and indirect.

That climate, in this large sense, acts energetically on the human constitution, is confirmed by too many centuries of observation, and is too generally accepted to need any array of proof. It is only with regard to special influences that I shall offer evidence.

The opinion which I have long held of the salubrity of the Northeast Plateau, has been fully confirmed by the minute information which I have obtained as supervisor of the census of North Georgia. But while I have a record of the mortality among both white and colored people, I shall confine myself mostly to that of the white population. For this two reasons exist.

The mortality of the two races is unequal. Both in freedom and slavery the mortality of the colored has been greater than that of the white population. And this is especially true in regard to pulmonary diseases; as is confirmed by the relative mortality of the white and colored troops of the British army.

This essay is written, also, only for the instruction of invalids seeking a more favorable climate, and especially for the white population of the North. In pointing out what may be their expectation of health and life, it is necessary to limit the statistics to those of the white race.

The reader will understand, therefore, that any statement of mortality for this region, unless otherwise specified, relates to the white race only.

The mortality of the whites in this region, outside of the city of Atlanta, was, for the year ending on the first of June, 1880, according to the census, in the ratio of ten deaths to the thousand people. This, for so large an area, and for as thorough work as I have reason to think was done by the enumerators of North Georgia, is small. It is conclusive as to the general healthfulness of the region.

The mortality of the City of Atlanta was, for the latter half of the year 1879, according to the report of the Board of Health, in the ratio of seven deaths to the thousand people, at the rate of fourteen to the thousand for a twelve month. This also is a very small city mortality.

It is worthy of observation that the latter half of the year includes the months that are usually most fatal in American cities. But I can testify from an examination of the mortuary report of Atlanta for five years, that the great increase of mortality which characterizes most American cities, Northern as well as Southern, does not occur in Atlanta, that the general health is better here during those months than in Northern cities.

Wherever malaria exists, there, other conditions being the same, is an increased ratio of mortality from consumption. I was, therefore, much pleased to find in the mortuary statistics of the census an almost entire absence of all forms of bilious fever, and of diseases of the liver in general. Typhoid fever occurs, as it does everywhere, even at nine thousand feet altitude on the Andes. But this is eminently an avoidable disease, due to neglect of proper sanitary precautions.

Pulmonary diseases are, in the temperate zones, far more fatal than those of any other class. And while pleurisy, bronchitis and other forms prove fatal, consumption and pneumonia cause the great mass of deaths from this class.

Consumption alone caused, in 1870, fourteen per cent. of the total mortality of the United States; consumption and pneumonia twenty two and one-third per cent. at the rate of 185 to every 100,000 of the population. In consequence of greater thoroughness in taking the census of this year, it is probable that in most sections both this ratio and that of the total mortality will be reported higher than for any previous year. According to the census of 1850, 1860 and 1870, consumption is at a maximum, and pneumonia at a minimum, throughout a belt stretching from the Atlantic to the Pacific, north of the forty-second parallel of latitude; while pneumonia is at its maximum, and consumption at its minimum, in a belt south of the thirty-fifth parallel, pleurisy usually having its maximum where pneumonia does. But bronchitis proves the most fatal in the middle belt, between the forty-second and thirty-fifth parallels, as is indicated by the censuses of 1850 and 1860.

I think it a fair induction from our mortality statistics, that, other conditions being equal, mortality from consumption increases, and mortality from pneumonia diminishes, with increase of latitude. In the North the more chronic, and in the South the more acute, pulmonary disease is the most prevalent.

In all the three censuses, there is a pretty uniform increase of mortality from consumption from North to South, in the country along the Atlantic coast, mortality from consumption for this region attaining to its maximum in New England, with its minimum in Florida, pneumonia attaining to its maximum in Florida, with its minimum in New England.

In consequence of less uniformity of conditions, some of them temporary, the progressive change with change of latitude is less marked on the Pacific coast and in the Mississippi Valley.

But I will venture to lay down, as a general rule, that consumption prevails, other conditions being the same, with cold and dampness, and pneumonia with heat and dampness. And since heat and cold are, in general, in relation to latitude, this becomes an index.

An attempt has been made to show that the prevalence of consumption has no connection with latitude, or with climatic conditions at all, by comparison of the mortality caused by it in cities North and South.

In order to illustrate this, I give below a table of the mortality for six months, from consumption in several cities, from data published in the Bulletin of the National Board of Health. I should have given it for a year if I had possessed a complete copy. To get the mortality for a year, it is only necessary to double the sums. When partial deficiences occurred I added a corresponding ratio.

The numbers express the ratio of mortality from consumption to 100,000 of the population.

*Mortality from Consumption of White and Colored.*

| | | | |
|---|---|---|---|
| Lowell, Mass............... | 173 | Baltimore, Md............. | 158 |
| Providence, R. I.......... | 134 | Norfolk, Va.................. | 180 |
| Brooklyn, N. Y............ | 158 | Charleston, S. C........... | 175 |
| Newark, N. J............... | 145 | Savannah, Ga.............. | 224 |

Apparently, it is plain that the prevalence of consumption has no relation to latitude.

Below I present another table, giving for 1870 the mortality from consumption for corresponding States. Let the reader bear in mind that, while in each table the ratio is to 100,000 of the population, it is for the following table for a year, and not for half a year as is the preceding one. But is is probable, since the mortality in the preceding table is derived from the burial permits of the cities, and that of the following one from the statistics of the census of 1870, that the former is complete and the latter incomplete. This, however, will not affect the ratios for latitude.

*Mortality from Consumption of White and Colored.*

| | | | |
|---|---|---|---|
| Massachusetts............... | 353 | Virginia......... ......... ... | 171 |
| Rhode Island................ | 254 | North Carolina............ | 115 |
| New York.................... | 264 | South Carolina............ | 93 |
| New Jersey.................. | 209 | Georgia.................. ...... | 75 |

It is evident, if in the cities of these States the mortality may be the same North and South, that outside of the cities the difference must be all the greater between North and South; and the legitimate conclusion is simply, that while climatic influences, connected with latitude, do tend to affect the distribution of consumption, other influences may be so potent as wholly to obscure them, a fact of which I have long been aware. And among these are the habits of life, and other influences characteristic of life.

For the same six months for which the estimate of the table of cities was made, the mortality from consumption in the city of Atlanta was for the whole population at the rate of 93 deaths to 100,000 people, 41 less than the lowest and 131 less than the highest rate of the table.

For the latter half of the year 1879, it was, according to the report of the Board of Health of Atlanta, 89 to the 100,000.

That this small ratio is not due to inland position merely, appears from a comparison with Augusta, Georgia, in which, during the six months first mentioned, the mortality from comsumption was in the ratio of 129 in the 100,000, being 36 greater than that of Atlanta.

In order to present to the reader these ratios in the most convenient form for reference, I add Augusta and Atlanta to the table of cities:

*Mortality from Consumption of White and Colored.*

| | | | |
|---|---|---|---|
| Lowell, Mass | 173 | Norfolk, Va | 180 |
| Providence, R. I | 134 | Charleston, S. C | 175 |
| Brooklyn, N. Y | 158 | Savannah, Ga | 224 |
| Newark, N. J | 145 | Augusta, Ga | 129 |
| Baltimore, Md· | 158 | Atlanta, Ga | 93 |

If any one should be disposed to attribute the small ratio in Atlanta to the rapid increase of population, I would remind him that Lowell and Newark have also been growing with remarkable rapidity.

If he thinks it to be due to an increase in Atlanta of a population less predisposed to consumption, I would remind him that a large proportion of the increase has been colored. During the latter half of 1879, for which the white mortality from consumption was, according to the Board of Health, in the ratio of 71 to the 100,000, that of the colored was 119, a smaller ratio, I presume, than will be found in any other cities where they exist in considerable numbers.

The ratio of mortality from pneumonia for the white population during the census year is also small, being as 86 to 100,000.

That the reader may the better appreciate the value of this ratio, I will add that in 1870 Georgia reported, on the same basis, a ratio of mortality from pneumonia of 115, Florida one of 144, Alabama one of 151, Mississippi one of 142, and Lousiana one of 178; the last exceeding by 40 per cent, that of Wisconsin from consumption, and being four times as great as that of Wisconsin from pneumonia.

In the damp, chilly air of New England is the maximum of consumption for the whole country. In the hot, humid air of Louisiana, laden with moisture from the Gulf, floating up the trough of the Mississippi and rising from swamps and bayous, is the maximum of mortality from pneumonia.

While consumption and pneumania are generally contrasted in point of prevalence in any given region, the regions with a high rate of mortality from pneumonia are not desirable places of residence for the consumptive. Consumption is characterized by inflammation and fever, and whatever tends to promote these, tends, also, to accelerate the progress of consumption when once established. The climate that is fruitful in pneumonia tends to aggrevate the inflamation and fever of the consumptive. The heat and humidity that develop the inflamation and fever of the subject of pneumonia, intensify the inflamation and fever of the consumptive. The hot, humid air of the Gulf Lowlands, also, contributes to relax still more the already enfeebled system of the consumptive. A residence in them renders the system all the more susceptible to rigorous weather or other noxious and morbific influences which he may encounter in the North. He usually shortens his life by his residence in Southern Lowlands.

What he needs is a climate which disposes in the least degree to both consumption and pnumonia. Such a one exists on the Northeast Plateau of Georgia.

To illustrate this I will give a table, which I have made up from the census of 1870, presenting the ratio to 100,000 people, of deaths from both consumption and pneumonia in those sections which are commended as desirable places of resort for consumptives:

*Aggregate Mortality of White and Colored from Consumption and Pneumonia.*

| | | | |
|---|---|---|---|
| California | 322 | Minnesota | 144 |
| Texas | 231 | Colorado | 128 |
| Florida | 213 | New Mexico | 118 |

And I have good reason to think, from the statistics of disease and death of United States troops at different stations, that the figures of this table fairly represent the chances of living for the consumptive, in general; the chances being in an inverse ratio to the numbers, the greater the number the less being the chance.

According to the census of 1880, the ratio of the aggregate mortality from these diseases was for the city of Atlanta only 205, and for the Northeast Plateau, outside of the city, only 115.

It should be remembered, also, in connection with a comparison between the census of 1870 and 1880, that it is probable the more thorough work of the latter year will raise the ratio of mortality from consumption and pneumonia in the States with which the comparison is made, and thus render it still more favorable to the Northeast Plateau, including Atlanta, for which region I have given the mortality according to the census of 1880

If it is urged that the spareness of the population and the habits of life of the people of this Highland region are particularly favorable to avoidance of consumption and pneumonia, I reply that it is much more densely peopled than New Mexico, and that the people of the Highland live out of doors less than do those of New Mexico, acknowledged by all who have studied the subject, to present the best chance for life to the consumptive of all our great civil divisions. But, so far as can be judged, the Northeast Plateau offers a better.

As to Atlanta. the habits of the people are like those of the people of other cities, neither more or less favorable, and yet for a city, it presents a remarkably low ratio of mortality from consumption and pneumonia.

There is one point in which a change of habit, in the city to some extent, and outside of the city to a very great extent, would cause a notable reduction of the reported mortality from pneumonia. The people of the Plateau have become so much accustomed to sitting with open doors and windows that they continue the practice when the weather demands a closed house. They often sit with one side warmed by a fire and the other chilled by a draft of wintry air, of all positions the most favorable for producing colds and coughs.

Adults may endure this with only a common cold as a frequent result, and pneumonia as an occasional one. But the infant, with its small heat-producing capacity, gets an attack of infantile bronchitis, and this is reported as pneumonia. My attention was constantly arrested in examining the one hundred and ninety-nine portfolios that came under my supervision, by the great number of cases of mortality from pneumonia of nursing babes, with whom it very rarely occurs. In several cases which I investigated, I found that the reported pneumonia was really capillary bronchitis, a disease especially pertaining to infancy; and

I have no doubt that nearly all the other cases of reported pneumonia in infants were of the same character. With a change of habit in regard to open doors and windows in inclement weather, infant mortality will be notably diminished.

Deduct the cases of infantile bronchitis reported as pneumonia, and the ratio of the latter becomes exceedingly small, so much so as to be in that respect without a parallel, perhaps, in the United States.

*Conclusion.*—So far as statistics indicate, no other so favorable place of resort for the consumptive is to be found in the whole country; and my experience in the treatment of patients here, goes to confirm the conclusion derived from statistics. I have had those who had tried Florida and other Gulf Lowlands before visiting this region, as well as those who had come directly from the North. A son, who has had extensive experience in the treatment of pulmonary diseases in Colorado, holds the same opinion of the merits of this region as I do. The subject of consumption formerly, he has tried Texas as well as Colorado. He finds himself in better health in this region than any other in which he has lived.

But while recommending this region, I would warn physicians against sending abroad consumptives who are on the verge of the grave. They usually die as soon as if they had remained at home, sometimes, I think, sooner, and without their last hours being soothed by the associations and comforts of home.

If, however, they will send those for whom there is hope, will send them, more especially, while the invalids do not as yet believe that they have consumption, while yet the physician hesitates to grieve them by giving them assurance that they are consumptives, I will guarantee that many will recover by a residence on the Northeast Plateau.

But, in order to do this, they need to place themselves under the care of physicians who have had ample experience in both the therapeutics and hygiene of consumption. When I insist on the importance of this, I insist on what I know, and what I could easily establish by the testimony of disinterested non-professional observers, invalids and others.

I know that many invalids fancy themselves too wise to need professional superintendence; that their native and untaught wisdom is of more value than that of a physician, however great his study, or however ample his experience. But the old adage in regard to lawyer and client applies, with a change of names to physician and patient. The invalid who is his own doctor generally has a fool for his patient. I do not wish the good reputation of the Plateau to suffer from becoming a resort for either hopeless or foolish invalids.

I would, also, beg every one who reads this tract, to remember that I invite invalids to no tropical climate, with its debilitating winter months, winter in name, but not in fact. I invite them to this region because it is not tropical, because it is cold enough to give tone to the enfeebled system without being severe enough to exhaust the small store of vitality. I have learned by observation that, for consumptives in general, a winter with a temperature below forty is not best, and that one above forty-seven is too warm for permanent improvement. But I would far rather advise a winter below forty in a dry climate, than one above forty-seven in a moderately humid one. During the unusually warm winter of 1879–80, with a mean temperature of 50°, notwithstanding the dryness of the air, invalids did not do as well on this Plateau as they did in colder winters.

Dr. Frederick Lente, who represented Florida on the Executive Committee of the Centennial Commission, says, in a eulogy on Florida as a place of resort for consumptives, "that when the temperature was in the eighties, he heard invalids complaining from day to day" * * * "was gratified when the mercury showed 69 at the same hour on the 16th, to hear their expressions of relief and to see their entire change of manner." One would think that Dr. Lente might have reasoned to the conclusion that a climate where the mercury does not climb into the eighties in the winter, might prove still more favorable to invalids, and that he might, without putting a great strain on his intellect, have asked himself the question, whether a temperature usually below 60 in the warmer part of the day, might not prove still more beneficial.

Long experience has taught me that most consumptives do best when the weather is cool enough (or nearly so,) for frost at night. Even those who are unduly sensitive to cold and who complain of it in such weather, acquire appetite and strength, and experience general improvement.

Without claiming for myself any superior native wisdom, I may, without presumption, say that I have studied this subject as few men have. I have had three cases of consumption in my own family, not pronounced so by myself alone, but by specialists of reputation in pulmonary diseases. I have myself suffered not a little from a formidable bronchitis, from lobular pneumonia and from pericardial effusion. I have had hectic fever, night sweats and muco-purent expectoration. I have had every motive to inform myself accurately as to the most desirable location for invalids with pulmonary diseases. And I express myself with great confidence, when I assert as the result of much study and many years' observation, that, in general, the subjects of pulmonary diseases, improve most in a region not where the ratio of mortality from consumption is least, but where the ratio of the aggregate mortality from both consumption and pneumonia is at a minimum.

So far as any accessible evidence teaches at the present time, this region is found on the Northeast Plateau of Georgia; and I again present it to the reader in a tabulated form, with the admonition that the considerable difference between Atlanta and the rural districts of the Plateau, is due, in considerable part, to avoidable causes, as I can establish by facts within my knowledge.

In order that the reader may take in at a glance the ratio borne by the aggregate mortality from consumption and pneumonia in Atlanta, and on the Plateau outside of Atlanta, to that of the various sections which have had a reputation for being sanitary for consumptives, I will add Atlanta and the rural districts of the Plateau to the table.

In giving in the table, the mortality for the whites only, for reasons previously assigned, I have taken into consideration, also, that the colored population of California, New Mexico, Colorado and Minnesota is too small to affect, appreciably, the ratio for those sections. And the high temperature of Texas and Florida, pernicious* to the white race, is so favorable to the colored race that it will go far towards equalizing the ratio for those States.

*Ratio to* 100,000 *people of aggregate deaths from consumption and pneumonia.*

| | | | |
|---|---|---|---|
| California | 322 | Minnesota | 144 |
| Texas | 231 | Colorado | 128 |
| Florida | 213 | New Mexico | 118 |
| Atlanta | 205 | Rural Plateau | 115 |

Time, with changed conditions, will modify the ratios of this table.

Regions sparsely peopled, like New Mexico, will exhibit a larger ratio when densely peopled; as will Colorado and Minnesota when the population shall no longer consist so largely of young or middle aged, and vigorous immigrants, all of which considerations apply in a considerable degree to Florida.

With allowance for the peculiar conditions of these sections, the ratios afford trustworthy indications.

N. B.—Invalids who may wish to reside either in the city or outside of it, can learn where to find desirable temporary or permanent homes by calling at No. 27½ Whitehall street, or by addressing the writer at the same place.

*So pernicious to the white race is a high, especially a protracted high, temperature, that the English residents in the East Indies cannot rear families there. And in our own country, the maximum mortality from all causes is in the extreme South, and the minimum in the extreme North; it being more than twice as great in the former as in the latter. While, as is well known, the black race enjoys the temperature that is so pernicious to the lighter colored.

It is to me a curious and interesting fact, that while the white race thrives best in a cool and the black in a hot climate, the intermediate colors, red and yellow, are equally vigorous throughout both zones, from the Equator to the Artic Circle.

